# Focal pericoronary adipose tissue attenuation is related to plaque presence, plaque type, and stenosis severity in coronary CTA

**DOI:** 10.1007/s00330-021-07882-1

**Published:** 2021-04-16

**Authors:** Runlei Ma, Marly van Assen, Daan Ties, Gert Jan Pelgrim, Randy van Dijk, Grigory Sidorenkov, Peter M. A. van Ooijen, Pim van der Harst, Rozemarijn Vliegenthart

**Affiliations:** 1 Department of Radiology, University Medical Center Groningen, University of Groningen, EB44, Hanzeplein 1, 9713 Groningen, GZ Netherlands; 2 Department of Radiology, Affiliated Hospital of Nanjing University of Chinese Medicine, Nanjing, China; 3 Department of Radiology and Imaging Sciences, Emory University School of Medicine, Emory University, Atlanta, GA USA; 4 Department of Cardiology, University Medical Center Groningen, University of Groningen, Groningen, Netherlands; 5 Department of Epidemiology, University Medical Center Groningen, University of Groningen, Groningen, Netherlands; 6 Department of Radiation Oncology, University Medical Center Groningen, Groningen, Netherlands; 7 Data Science Center in Health (DASH), University Medical Center Groningen, Groningen, Netherlands

**Keywords:** Computed tomography angiography, Atherosclerosis, Adipose tissue, Coronary arteries

## Abstract

**Objectives:**

To investigate the association of pericoronary adipose tissue mean attenuation (PCAT_MA_) with coronary artery disease (CAD) characteristics on coronary computed tomography angiography (CCTA).

**Methods:**

We retrospectively investigated 165 symptomatic patients who underwent third-generation dual-source CCTA at 70kVp: 93 with and 72 without CAD (204 arteries with plaque, 291 without plaque). CCTA was evaluated for presence and characteristics of CAD per artery. PCAT_MA_ was measured proximally and across the most severe stenosis. Patient-level, proximal PCAT_MA_ was defined as the mean of the proximal PCAT_MA_ of the three main coronary arteries. Analyses were performed on patient and vessel level.

**Results:**

Mean proximal PCAT_MA_ was −96.2 ± 7.1 HU and −95.6 ± 7.8HU for patients with and without CAD (*p* = 0.644). In arteries with plaque, proximal and lesion-specific PCAT_MA_ was similar (−96.1 ± 9.6 HU, −95.9 ± 11.2 HU, *p* = 0.608). Lesion-specific PCAT_MA_ of arteries with plaque (−94.7 HU) differed from proximal PCAT_MA_ of arteries without plaque (−97.2 HU, *p* = 0.015). Minimal stenosis showed higher lesion-specific PCAT_MA_ (−94.0 HU) than severe stenosis (−98.5 HU, *p* = 0.030). Lesion-specific PCAT_MA_ of non-calcified, mixed, and calcified plaque was −96.5 HU, −94.6 HU, and −89.9 HU (*p* = 0.004). Vessel-based total plaque, lipid-rich necrotic core, and calcified plaque burden showed a very weak to moderate correlation with proximal PCAT_MA_.

**Conclusions:**

Lesion-specific PCAT_MA_ was higher in arteries with plaque than proximal PCAT_MA_ in arteries without plaque. Lesion-specific PCAT_MA_ was higher in non-calcified and mixed plaques compared to calcified plaques, and in minimal stenosis compared to severe; proximal PCAT_MA_ did not show these relationships. This suggests that lesion-specific PCAT_MA_ is related to plaque development and vulnerability.

**Key Points:**

*• In symptomatic patients undergoing CCTA at 70 kVp, PCAT*_*MA*_ *was higher in coronary arteries with plaque than those without plaque.*

*• PCAT*_*MA*_ *was higher for non-calcified and mixed plaques compared to calcified plaques, and for minimal stenosis compared to severe stenosis.*

*• In contrast to PCAT*_*MA*_ *measurement of the proximal vessels, lesion-specific PCAT*_*MA*_ *showed clear relationships with plaque presence and stenosis degree.*

**Supplementary Information:**

The online version contains supplementary material available at 10.1007/s00330-021-07882-1.

## Introduction

Coronary inflammation plays an important role in atherosclerosis development [1–3]. Detection and quantification of coronary inflammation could assist in early risk stratification of coronary artery disease (CAD) patients, possibly even before the development of coronary plaque [4]. Recently, a non-invasive biomarker for coronary inflammation was proposed: computed tomography angiography (CCTA) derived pericoronary adipose tissue mean attenuation (PCAT_MA_) [5]. PCAT_MA_ has shown value as a predictor for cardiac mortality [6]. Few studies, predominantly using the proximal right coronary artery (RCA) as a representative location for patient-level analysis, have shown a relationship of PCAT_MA_ with CAD and atherosclerosis progression [5, 7–9].

CCTA-based plaque composition and stenosis severity give information about plaque vulnerability and hemodynamic significance, and can be used for prognostication [10–13]. A previous study showed a PCAT_MA_ difference of 3–4HU in the proximal RCA between CAD and non-CAD patients [5]. However, they found no significant difference of RCA-based PCAT_MA_ between non-calcified plaques (NCP) and mixed or calcified plaques (CP) in patients with high plaque burden. Another study demonstrated that increased NCP and total plaque burden were associated with higher PCAT_MA_ [8].

Most studies measured PCAT_MA_ at one proximal coronary location [5, 6, 8, 14]. Compared to proximal PCAT_MA_, there may be a stronger relation of lesion-specific PCAT_MA_ with plaque considering a hypothesized local effect of coronary inflammation. Three PCAT_MA_ studies (35–199 patients) used a lesion-based measurement method considering all three main coronary arteries [9, 15, 16]. One study showed that lesion-specific PCAT_MA_ was higher around culprit lesions in acute coronary syndrome (ACS) patients compared to non-culprit lesions in ACS and CAD patients [15]. Another study revealed lesion-specific PCAT_MA_ was significantly increased in patients with abnormal FFR [9]. However, lesion-specific PCAT_MA_ failed to show a significant difference between patients with and without elevated high-sensitivity C-reactive protein [16]. Currently, there is a lack of knowledge on the relationship between PCAT_MA_ and plaque presence, plaque type, and stenosis severity. In addition, the majority of studies only investigated a single, proximally measured PCAT_MA_ value (mostly RCA) to represent overall pericoronary attenuation but did not investigate a potentially more relevant, focal PCAT_MA_ value across coronary plaque.

The aim of this study was to evaluate the relationship of proximal and lesion-specific PCAT_MA_ with coronary plaque presence, type, and severity.

## Materials and methods

### Study population

This single-center, cross-sectional study was performed at the University Medical Center Groningen. The study was compliant with the Declaration of Helsinki and approved by the institutional ethical review board, who waived the need for informed consent.

**Fig. 1:**
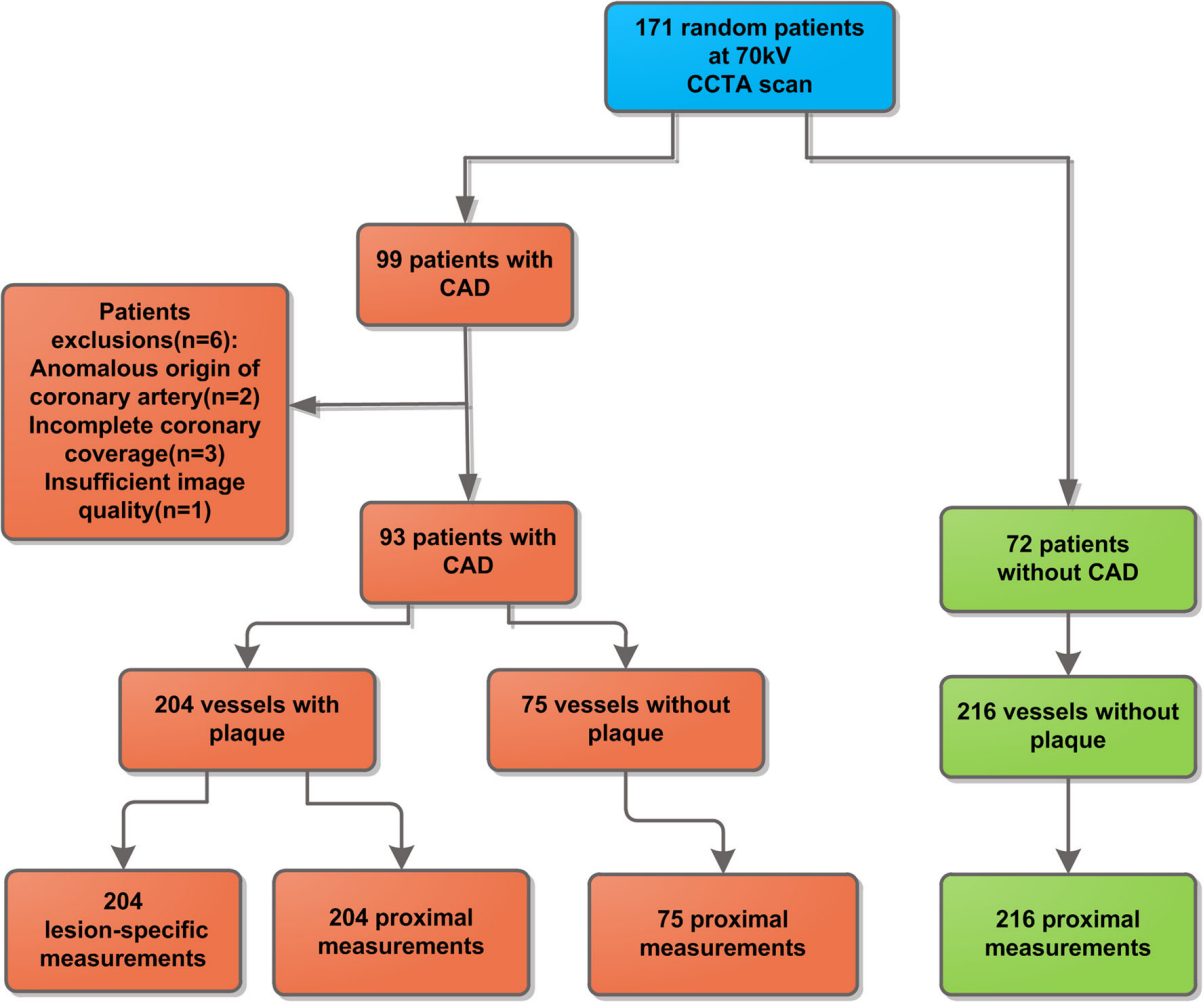
Flowchart of patient inclusion and PCAT_MA_ measurement analysis levels. kV is kilovoltage; CCTA is coronary computed tomography angiography; CAD coronary artery disease

In total, 2621 patients underwent cardiac CTA for routine indications between January 2015 and November 2017. Of these patients, a random sample of 1280 patients was further characterized by gathering hospital record information on CT indication, demographics, and clinical risk factors, to be used in various CT analyses. In a previous analysis (Ma et al) [17], we studied a cohort of patients with a zero calcium score and no coronary plaque on CCTA (“normal patients”); from this population, we selected patients with CCTA at 70 kilovoltage peak (kVp) as a reference category for the current study (*n* = 72). From the 697 patients (out of 1280) who underwent CCTA because of angina, we randomly selected patients with CAD, defined as patients with plaque on their CCTA images, for the current analysis based on the following inclusion criteria: 1, age > 18 years; 2, CCTA performed at 70 kVp; 3, no coronary stents or coronary artery bypass grafts. Tube voltage was restricted to 70 kVp in view of known influence of kVp on PCAT_MA_ [17]. In total, 171 patients (72 + 99) were included. Six CAD patients were excluded for the following reasons: anomalous origin of coronary artery (*n* = 2), insufficient image quality (*n* = 1), incomplete coronary image coverage (*n* = 3) (Fig. 1). A radiologist with 10-year experience in cardiac radiology performed the CCTA evaluation (R.M.). In case of doubt, a radiologist with 14 years of experience was consulted and consensus was obtained (R.V.).

### CCTA scan protocol

CCTA imaging was performed according to the routine clinical protocol using third-generation dual-source CT (SOMATOM Force, Siemens Healthineers). First, a non-enhanced ECG-gated CT at a high pitch (tube voltage 120 kVp, reference tube current 64 mAs, reconstructed slice thickness 3.0mm) was performed for coronary calcium score (CACS) analysis. Subsequently, CCTA was performed using CarekV (kVp optimization assistance), depending on patient size; patients scanned at 70 kVp were included. ECG-gated high-pitch spiral scanning was performed in low, regular heart rate, otherwise ECG-triggered sequential scanning. Patients received sublingual nitroglycerin, unless contraindicated. If the heart rate was > 70–73 beats/min, the patient received intravenous beta-blocker, unless contraindicated. Contrast timing was determined using a test bolus. Iomeprol (Iomeron 350) was injected with dose- and flow-rate depending on patient characteristics and scan mode. A dual-injection technique was used followed by a saline flush. CCTA images were reconstructed at 0.6 mm thickness.

### Patient characteristics

Baseline patient characteristics were collected from clinical records. Age, sex, and CAD risk factors were collected. The classification criteria of risk factors were as follows: (a) hypertension—systolic blood pressure > 140 mmHg or diastolic blood pressure > 90 mmHg according to guidelines [18] and/or anti-hypertension medication use; (b) hyperlipidemia—patients with a low-density lipoprotein > 4.5 mmol/L or total cholesterol > 6.5 mmol/L based on guidelines [19] were considered as hyperlipidemic; lipid-lowering medications used at the time of CT scanning was considered as a separate factor indicating treated hyperlipidemia; (c) diabetes mellitus—anti-diabetic medication use; (d) smoking status was classified as non-smoker, current smoker, or former smoker. Depending on the risk factors, information was missing in 26 to 51 patients. If there was no mention of a risk factor, the risk factor was considered absent. Body mass index (BMI) information was collected as well.

### Plaque analysis

#### Visual, qualitative analysis

For visual plaque evaluation only, the main coronary arteries, left anterior descending (LAD), left circumflex (LCx), and right coronary artery (RCA) were taken into account to optimize patient comparability. Plaque composition and diameter stenosis (DS) were assessed for the most severe plaque per coronary artery. Plaque components were classified into non-calcified plaque (NCP), mixed plaque, and calcified plaque (CP). Using visual analysis, CP was defined as plaque when it had > 75% volume with density higher than the luminal contrast; NCP was defined as plaque when it had > 75% volume with a density lower than the lumen contrast and higher than soft tissues around. Mixed plaque was defined as plaque comprising 25 to 75% volume with density higher than the luminal contrast [20, 21]. DS was classified into 4 stenosis categories: minimal, DS 1–24%; mild, DS 25–49%; moderate, DS 50–69%; and severe, DS 70–100% [22].

#### Quantitative analysis

Semi-automated software (Aquarius iNtuition, TeraRecon, Version 4.4.13) was used to measure the Agatston-based CACS on a per-patient level. The CACS was stratified into four categories: 0, 1–99, 100–399, and ≥ 400.

Quantification of the plaque composition was semi-automatically performed by the software (vascuCAP, Research Edition, Elucid Bioimaging) [23]. Automatic segmentation of the entire coronary lumen and wall was performed, allowing manual corrections if needed. Subsequently, the matrix burden, CP burden, and lipid-rich necrotic core (LRNC) burden were automatically calculated by the software on a per-vessel level [24]. The classification of the different plaque components, which was validated with plaque histology, was based on an adaptive threshold. The LRNC lower limit was defined as −300HU; LRNC-IPH boundary was defined as 25HU. The lower limit and upper limit of the CP were 250 and 3000HU. Matrix burden was calculated by dividing the total wall volume by the matrix volume, where the matrix is defined as normal organization tissues in the vessel wall [23]. Plaque burden was defined as 1-matrix burden [24].

### PCAT_MA_ measurements

**Fig. 2:**
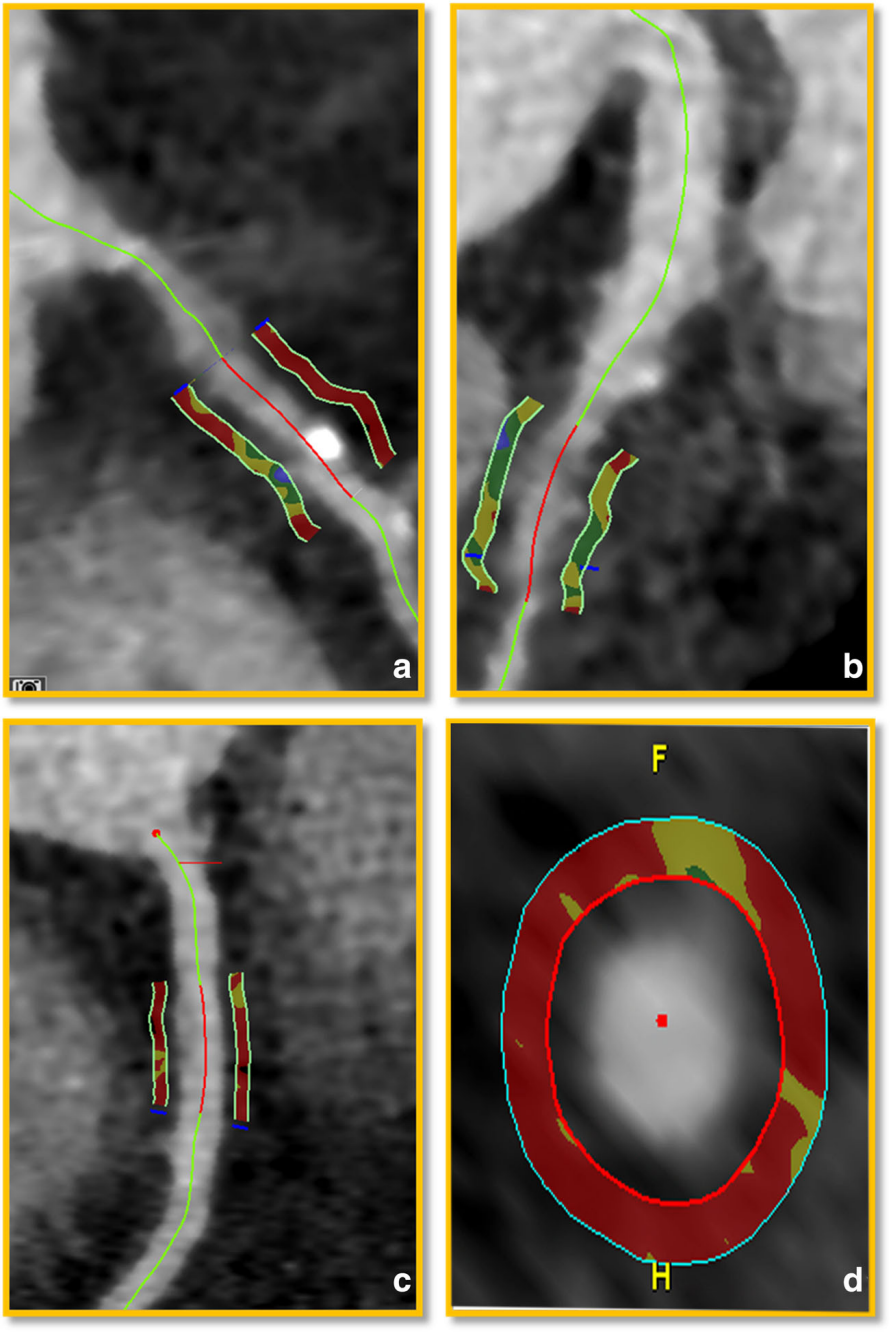
PCAT_MA_ measurements. **a** and **b** represent CCTA images from a 59-year-old male patient with CAD. **a** represents the lesion-specific PCAT_MA_ measurement in the RCA across a calcified plaque. **b** shows the lesion-specific PCAT_MA_ measurement across a non-calcified plaque in LAD. **c** and **d** represent CCTA images from a 56-year-old male patient without plaque. **c** shows the proximal PCAT_MA_ measurement of the RCA. **d** shows the cross-sectional view of the proximal PCAT_MA_ measurement in the RCA. The red zones indicate the areas used for PCAT_MA_ measurement

PCAT_MA_ was measured proximally in the RCA, LAD, and LCx, using dedicated software (Aquarius iNtuition, TeraRecon, Version 4.4.13). The starting point of the proximal PCAT_MA_ measurement was 10mm after the left main bifurcation for LAD, at the bifurcation point for LCx, and 10mm after the ostium for RCA [17]. In vessels with plaque, a lesion-specific PCAT_MA_ measurement was performed centered around the most severely stenotic plaque. The proximal and distal ends of the measurement were 5mm away from the lesion center. The measurement length and width for all measurements were 10mm and 1mm. A 1mm gap was left between the outer vessel wall, taking into account eccentric plaques, and the measured cylindrical volume to avoid artifacts. PCAT_MA_ was defined as the mean CT value in the measured area within the range of −190 to −30 HU (Fig. 2).

### Data analysis

First, PCAT_MA_ was studied on per-patient level (Fig. 1). Patients with any coronary plaque were considered as CAD patients; patients without plaque were considered non-CAD patients. For the per-patient PCAT_MA_, the mean of the proximal PCAT_MA_ values based on the three main coronary arteries was calculated to represent an overall, patient-based PCAT_MA_ value. Patient-based CACS and DS were analyzed in conjunction with the per-patient PCAT_MA_. Patient-level categorization of DS degree was based on the most severe DS in all three coronary arteries. To allow comparison with prior studies that used only the proximal measurement of PCAT_MA_ of the RCA, we additionally performed analyses for RCA-based PCAT_MA_. Additionally, a comparison of patients with and without at least 50% stenosis was performed. The total plaque burden of the main coronary arteries was considered as the patient-based plaque burden.

Second, vessel-based analysis was performed (Fig. 2). We discriminated arteries with any plaque, and arteries without plaque. CAD patients could contribute arteries without plaque. For arteries with multiple plaques, the lesion with the highest DS was used. The proximal PCAT_MA_ was used in arteries without plaque to compare with lesion-specific PCAT_MA_ in arteries with plaque. Lesion-specific PCAT_MA_ was analyzed based on plaque type and DS severity.

### Statistical methods

Normality testing for continuous variables was performed with the Shapiro-Wilk test. Continuous variables are represented as mean± standard deviation (SD) or median (interquartile range [IQR]), according to distribution. The model estimated values are given in mean with 95% confidence interval (CI). Categorical variables were recorded as numbers (*n*) and percentages (%). Paired *t*-tests were used to evaluate differences between proximal and lesion-specific PCAT_MA_. Independent *t*-tests were used to compare PCAT_MA_ measurements between patients. One-way analysis of variance (ANOVA) testing was used to compare PCAT_MA_ between categories of plaque type and DS severity. Spearman correlation testing was used to assess the correlation of PCAT_MA_ with plaque burden and plaque component burden.

A generalized linear model was used to evaluate the influencing factors for patient-based PCAT_MA_. Using mixed models with random intercepts, the model estimated marginal means and 95% CI of the corrected PCAT_MA_ were calculated. The basic model included age, sex, and vessel, while the advanced models included CAD risk factors. The models did not include BMI because of 43 missing values. PCAT_MA_ was taken as a dependent variable in order to study the relationship between PCAT_MA_ and plaque features. A *p* value < 0.05 was considered statistically significant. Statistical analyses were performed using SPSS version 25 (IBM).

## Results

### Patient demographics

In total, 93 patients with CAD and 72 patients without CAD were included. Figure 2 shows an overview of the inclusion process. Patient characteristics are given in Table 1. Patients with CAD were significantly older (60.9 ± 8.7 vs. 51.2 ± 12.6 years, *p* < 0.001) and had significantly more hypertension (39 [41.9%] vs. 16 [22.2%], *p* = 0.008) and hyperlipidemia (39 [41.9%] vs. 12 [16.7%], *p* < 0.001) compared to patients without CAD.

**Table 1:** Patient characteristics

Variables	CAD patients	Non-CAD patients	*p* value
*n*	93	72	
Male, *n* (%)	43 (46.2%)	23 (31.9%)	0.063
Age (years) (SD)	60.9 ± 8.7	51.2 ± 12.6	< 0.001
BMI (kg/m^2^) (SD)*	24.2 ± 2.9	23.2 ± 3.1	0.092
Hypertension, *n* (%)	39 (41.9%)	16 (22.2%)	0.008
Diabetes mellitus, *n* (%)	10 (10.8%)	3 (4.2%)	0.119
Hyperlipidemia, *n* (%)	39 (41.9%)	12 (16.7%)	< 0 .001
Statin use, *n* (%)	23 (24.7%)	6 (8.3%)	0.005
Smoking, *n* (%)			0.144
Former smoker	22 (23.7%)	8 (11.1%)	
Current smoker	26 (28.0%)	18 (25.0%)	
Family history of CAD, *n* (%)	41 (44.1%)	22 (30.6%)	0.076
Indication for CCTA, *n* (%)			0.517
Typical angina	12 (12.9%)	8 (11.1%)	
Atypical angina	50 (53.8%)	36 (50%)	
Non-anginal chest pain	2 (2.2%)	7 (9.7%)	
Dyspnea/dyspnea d’ effort	7 (7.5%)	5 (6.9%)	
Others*	22 (23.7%)	16 (22.2)	

BMI body mass index; SD standard deviation; CCTA coronary computed tomography angiography. BMI information was available for 122 patients. *Others included arrhythmias or high-risk profile

### Patient-based PCAT_MA_ analysis

An overview of PCAT_MA_ values for CAD and non-CAD patients, CACS, and DS category is provided in Table 2. There was no correlation between PCAT_MA_ and CACS (*r* = −0.006, *p* = 0.939). Correlation of PCAT_MA_ with DS category and plaque burden was very weak (*r* = 0.073, *p* = 0.486 and *r* = −0.092, *p* = 0.383). When corrected for age and sex, PCAT_MA_ showed no difference between patients with and without CAD (−95.7 HU vs −95.6 HU, *p* = 0.933). PCAT_MA_ was significantly different between sexes (men: −94.0 HU vs. women: −97.3 HU, *p* = 0.007). Results for proximal RCA-based PCAT_MA_ are provided in Table S1 and Table S2.

**Table 2:** PCAT_MA_ by CAC score and degree of stenosis, per-patient analysis

Patient-level evaluation	Mean proximal PCAT_MA_	*p* value
		0.325
CAC score 0	−95.4 ± 7.9 HU (*n* = 78)	
CAC score 1–99	−96.9 ± 7.1 HU (*n* = 35)	
CAC score 100–399	−97.3 ± 5. 7HU (*n* = 34)	
CAC score > 400	−94.0 ± 8.5 HU (*n* = 18)	
		0.644
no CAD	−95.6 ± 7.8 HU (*n* = 72)	
With CAD	−96.2 ± 7.1 HU (*n* = 93)	
		0.825
DS < 50%	−96.0 ± 7. 3HU (*n* = 121)	
DS ≥ 50%	−95.7 ± 7.6 HU (*n* = 44)	
		0.580
DS 1–24%	−98.3 ± 6.5 HU (*n* = 16)	
DS 25–49%	−95.8 ± 6.7 HU (*n* = 33)	
DS 50–69%	−94.9 ± 6.8 HU (*n* = 16)	
DS 70–100%	−96.2 ± 8.2 HU (*n* = 28)	

DS diameter stenosis; CAC coronary artery calcium; PCAT_MA_ pericoronary adipose tissues mean attenuation

### Vessel-based proximal PCAT_MA_ analysis

There were 204 arteries with plaque and 291 without plaque (216 from patients without CAD and 75 from patients with CAD). The mean proximal PCAT_MA_ of vessels without plaque was −95.6 ± 9.6 HU and −96.3 ± 8.3 HU for patients with and without CAD, respectively (*p* = 0.567). The different plaque components or degrees of stenosis groups did not show a difference in proximal PCAT_MA_.

### Vessel-based lesion-specific PCAT_MA_ analysis

Lesion-specific PCAT_MA_ showed a significant difference (*p* = 0.002) for the coronary lesions with different plaque components. However, there was no significant difference in degrees of stenosis (*p* = 0.288). In arteries with plaque (*n* = 204), the median [IQR] plaque burden was 32.9% [29.6–37.5%], showing a weak correlation with PCAT_MA_ (*r* = −0.260, *p* < 0.001). The median LRNC plaque burden was 9.9% [5.9–13.7%], showing a moderate correlation with PCAT_MA_ (*r* = −0.325, *p* < 0.001). Median CP burden was 4.1% [1.9–7.9%], with a weak correlation between PCAT_MA_ and CP burden (*r* = −0.097, *p* = 0.167).

**Fig. 3:**
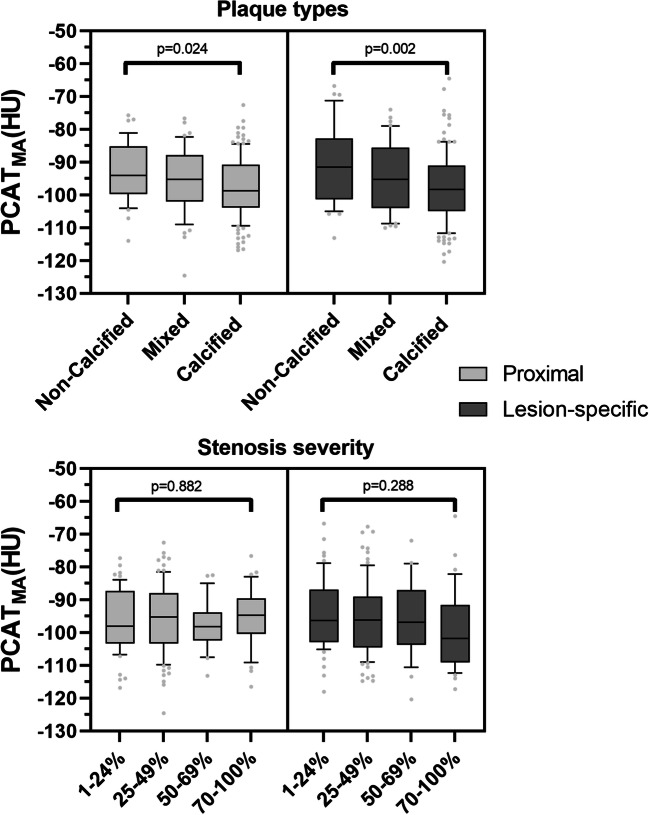
Proximal and lesion-specific PCAT_MA_ by plaque type and stenosis severity. PCAT_MA_ pericoronary adipose tissue mean attenuation

Figure 3 gives an overview of proximal and lesion-specific PCAT_MA_ measurements for different plaque components and degrees of stenosis.

### Model-based analysis of PCAT_MA_

In the basic model, the corrected mean (95% CI) PCAT_MA_ was −94.1 HU (−95.7; −92.5 HU) in vessels with plaque (lesion-specific) and −96.3 HU −97.8; −94.9 HU) in vessels without plaque in non-CAD patients (proximal) (*p* = 0.026) (Table 3). Sex (*p* = 0.032), age (*p* = 0.018), and vessel (LAD, LCx, RCA) had significant effects on PCAT_MA_ (*p* < 0.001). The mean (95% CI) lesion-specific PCAT_MA_ of NCP, mixed, and CP was −90.2 HU (−93.8; −86.7 HU), −94.8 HU (−98.0; −91.6 HU), and −96.6 (−98.6; −94.5 HU), respectively (*p* = 0.006). For DS categories, the overall group effect did not reach statistical significance (*p* = 0.073), but PCAT_MA_ of severe DS was significantly different from minimal DS (*p* = 0.037). For the advanced models, including CAD risk factors, the differences remained significant (Table 3). For the model with all healthy and diseased vessels, there was a significant difference of PCAT_MA_ between patients with and without statin use (−97.6 HU vs −94.3 HU, *p* = 0.039). Table S3 shows results comparing proximal PCAT_MA_ between plaque types and DS using all arteries with and without plaque combined.

**Table 3:** Mixed linear models for PCAT_MA_ and plaque characteristics

Categories	Basic models			Advanced models		
	Estimated fixed effect (95% CI)	Estimated mean (95% CI) (HU)	*p* value	Estimated fixed effect (95% CI)	Estimated mean (95% CI) (HU)	*p* value
Models of vessels with and without plaque						
Vessels without plaque	0 (Ref)	−96.3 (−97.8; −94.9)	0.026*	0 (0)	−97.2 (−100.0; −94.3)	0.015*
Vessels with plaque	3.7 (1.0; 6.4)	−94.1 (−95.7; −92.5)	0.026	3.9 (1.2;6.7)	−94.7 (−97.5; −92.0)	0.015
Models of vessels with plaque						
*Type of plaque*						
Non-calcified (*n* = 38)	4.5 (−0.6; 9.7)	−90.2 (−93.8; −86.7)	0.001	4.7 (−0.5;9.8)	−89.9 (−94.3; −85.4)	0.001
Mixed (*n* = 45)	1.3 (−4.3; 6.8)	−94.8 (−98.0; −91.6)	0.329	0.9 (−4.6; 6.5)	−94.6 (−98.6; −90.5)	0.301
Calcified (*n* = 121)	0 (Ref)	−96.6 (−98.6; −94.5)	0.006*	0(Ref)	−96.5 (−99.8; −93.2)	0.004*
*Degree of stenosis*						
1–24% (*n* = 59)	0 (Ref)	−94.4 (−97.2; −91.6)	0.073*	0 (Ref)	−94.0 (−97.9; −90.1)	0.079*
25–49% (*n* = 85)	0.5 (−4.5; 5.5)	−94.1 (−96.5; −91.7)	0.856	0.2 (−4.8; 5.2)	−93.8 (−97.6; −90.1)	0.927
50–69% (*n* = 26)	5.0 (−5.1; 15.1)	−93.2 (−97.5; −88.8)	0.622	3.7 (−6.5; 13.8)	−93.3 (−98.4; −88.3)	0.798
70–100% (*n* = 34)	−3.5 (−10.2; 3.2)	−98.8 (−102.2; −95.3)	0.037	−3.6 (−10.3; 3.1)	−98.5 (−102.9; −94.1)	0.030
*Plaque component burden*						
LRNC burden	−0.8 (−1.2; 0.4)		0.009	−0.7 (−1.1; −0.3)		0.014
Calcified plaque burden	−0.3 (−0.9; 0.3)		0.326	−0.3 (−0.9; 0.3)		0.336
Plaque burden	−0.6 (−1.0; −0.2)		0.003	−0.6 (−1.0; −0.2)		0.007

CAD coronary artery disease; CI confidence interval; HU Hounsfield unit; PCATMA pericoronary adipose tissues mean attenuation; LRNC lipid-rich necrosis core. Values are lesion-specific PCAT_MA_ values, apart from vessels without plaque (proximal PCAT_MA_). * is the fixed effect *p* value of the factor

After correction for CAD risk factors, LRNC burden and plaque burden had significant effects (estimate: −0.8 vs. −0.6) on proximal PCAT_MA_, while the CP burden had no significant effects on proximal PCAT_MA_ (Table 3).

## Discussion

This study investigated the relationship between PCAT_MA_ and plaque presence, plaque type, and stenosis severity in the main coronary arteries in symptomatic patients undergoing CCTA at 70 kVp. PCAT_MA_ was higher in vessels with plaque than in vessels without plaque, taking into account patients’ risk factors. Lesion-specific PCAT_MA_ was higher for non-calcified and mixed plaques compared to calcified plaques, and for minimal stenosis compared to severe stenosis. In contrast to proximal PCAT_MA_, lesion-specific PCAT_MA_ showed clear relationships with plaque presence and stenosis degree.

The proof-of-concept paper by Antonopoulos et al [5] demonstrated that RCA-based PCAT_MA_ differed by approximately 3HU between CAD and non-CAD patients, where CAD was defined as the presence of a stenosis of more than 50%. As PCAT_MA_ values vary between coronary arteries and plaque distribution among the coronary arteries, with the LAD most often affected, taking only the RCA as a PCAT_MA_ reference location may not accurately represent the patient’s PCAT_MA_ status. Oikonomou et al [6] reported that increased PCAT_MA_ in the RCA and LAD rather than LCx was related to increased cardiac mortality risk. Gaibazzi et al [25] reported significant differences between the LAD/RCA and the LCX in vessels with a stenosis < 50%, with a HU difference of approximately 1.5 HU on 120kVp scans. In our previous study, comparing PCAT_MA_ at different kVp levels in patients without plaque, there were significant differences between the PCAT_MA_ of LAD, LCX, and RCA with a HU difference around 2~4 HU [17].

Besides the coronary artery, the measurement location may also have a significant effect on PCAT_MA_. Goeller et al [8] showed that, although there was a correlation between PCAT_MA_ and epicardial adipose tissue (EAT), there was no correlation between changes in EAT and plaque burden progression. Dai et al [16] found no relationship between lesion-specific PCAT_MA_ and high-sensitive C-reactive protein, suggesting that PCAT_MA_ may be associated with local coronary inflammation rather than global inflammation. Previously mentioned studies used lesion-specific PCAT_MA_ only; few investigated the relationship with coronary plaque. Kwiecinski et al [26] found that increased lesion-specific PCAT_MA_ in patients with high-risk plaque was related to focal 18F-NaF PET uptake. Lin et al [27] reported on the relationship of PCAT radiomic features and PCAT_MA_ in the proximal RCA and around (non-) culprit lesions at presentation and 6 months post-MI, in comparison to stable CAD and non-CAD cases. They report that the most significant radiomic parameters distinguishing patients with and without MI were based on texture and geometry, yielding information not included in PCAT attenuation. They found that radiomic features were not different between culprit and non-culprit lesions, where the PCAT_MA_ showed a significant difference. The authors mention that PCAT_MA_ may have utility as a lesion-specific imaging biomarker, while radiomics features may have more value as a patient-specific biomarker of systemic inflammation. Our study, using both proximal and lesion-based PCAT_MA_, confirms that lesion-specific PCAT_MA_ is a better representation of focal inflammation and plaque development. Only lesion-specific PCAT_MA_ measurements showed a difference between vessels with and without plaque. Using an adjusted model, the PCAT_MA_ of vessels with plaque was around 2HU higher than those without plaque. This result is similar to the HU difference in the study by Antonopoulos et al [5].

Lesion-specific PCAT_MA_ differed by DS categories, taking into account age, sex, and coronary artery. Our results suggest that there may be more inflammation in mild and moderate DS than in severe DS. This fits with the hypothesis that as the plaque becomes more stabilized and more calcified in severe DS, inflammation could be relatively decreased [28]. Inflammatory cytokines play a critical role in the development and progression of coronary atherosclerosis [29, 30]. The theory behind PCAT_MA_ is that vessel wall atherosclerosis inhibits adipocyte maturation and lipid accumulation in the pericoronary fat tissue, increasing the attenuation. Additionally, corresponding increases in edema and amount of inflammatory cells possibly result in an additional increase in PCAT_MA_ in patients at risk of or with CAD [31, 32]. Results from previous studies suggest that the relationship between coronary inflammation and PCAT_MA_ may be more evident in NCP than CP, since CPs are relatively stable and have only a minimal inflammatory component [31, 32]. Goeller et al [8] investigated the relationship between PCAT_MA_ and progression of plaque burden on CCTA. Measuring patient-based plaque burden/composition and RCA-based PCAT_MA_, they found that PCAT_MA_ is related to progression of total plaque burden and NCP burden. PCAT_MA_ > −75 HU of the proximal RCA was independently associated with increased NCP burden at 120kVp CCTA [8]. However, similar to our results, they found that there was no relationship with CP burden. In our study, the model-adjusted, lesion-specific PCAT_MA_ values for NCP were 5–7 HU higher compared to CP and mixed plaques at 70kVp CCTA, measured in the three main coronary arteries. Our study showed only a weak correlation between vessel-based plaque burden and per-vessel PCAT_MA_, and no significant correlation between patient-based total plaque burden and patient-based PCAT_MA_. The per-vessel LRNC burden had a moderate correlation with PCAT_MA_ whereas the CP burden showed a very poor correlation. Recent research revealed that LRNC burden is capable of predicting myocardial infarction better than CAC scoring, cardiovascular risk scores, and coronary artery stenosis [33].

There are reports that show that lipid-lowering medication could decrease the EAT attenuation independent of decreasing lipid values [34]. Our study also shows a significant effect of lipid-lowering medication on PCAT_MA_ values, supporting the idea that statins have an effect on cardiac fat attenuation and, potentially, adipose tissue activity [35]. Additionally, we found that vessel, sex, and age had significant effects on PCAT_MA_. The relationship between age, sex, and CAD has been reported frequently [36–38]. Men showed generally higher PCAT_MA_ values than women (−94.0 vs −97.3 HU). Gender-specific hormones may be the reason for the different effects on coronary inflammation.

## Limitations

This is a single-center, cross-sectional study of patients with clinically indicated CCTA. No follow-up information is available; hence, CCTA results cannot be related to cardiovascular prognosis. Although our study demonstrates a relationship between plaque presence, type, and stenosis degree with PCAT_MA_, it was not designed to show direct causality between inflammatory status, plaque characterization, and PCAT_MA_. Plaque burden quantification was performed by automatic software, allowing manual corrections. In general, automatic analysis might be sensitive to errors due to image artifacts or decreased image quality and errors in segmentation. To avoid these errors in this study, scans were selected on image quality (2 scans were excluded), and at each segmentation step, the segmentation was visually assessed and manually corrected when necessary by an experienced radiologist to avoid errors. Window levels could be adjusted manually to reduce, for example, blooming effects from calcifications in order to optimize the segmentation and automated analysis.

## Conclusion

PCAT_MA_ was higher in coronary arteries with plaque, compared to vessels without plaque. Lesion-specific PCAT_MA_ was higher in NCP and mixed plaque compared to CP, and in minimal stenosis compared to severe stenosis. Proximally measured PCAT_MA_ only showed differences by plaque composition, and only when corrected for clinical parameters. This suggests that in particular lesion-specific PCAT_MA_ is related to plaque development and vulnerability.

## Supplementary Information

ESM 1 (DOCX 23 kb)

## Abbreviations

BMI: Body mass index
CAD: Coronary artery disease
CCTA: Coronary computed tomography angiography
CP: Calcified plaque
DS: Diameter stenosis
ICC: Intra-class correlation coefficient
IQR: Interquartile range
kVp: Kilovoltage peak
LAD: Left anterior descending coronary artery
LCx: Left circumflex coronary artery
LRNC: Lipid-rich necrotic core
NCP: Non-calcified plaque
PCAT_MA_: Pericoronary adipose tissue mean attenuation
RCA: Right coronary artery
SD: Standard deviation
