# Supplementary material for: Focal pericoronary adipose tissue attenuation is related to plaque presence, plaque type, and stenosis severity in coronary CTA
Source: Eur Radiol. 2021 Apr 16;31(10):7251–61. doi: 10.1007/s00330-021-07882-1 (PMC8452552; doi:10.1007/s00330-021-07882-1)
Supplement: Supplementary file 1 — (DOCX 23 kb) [file 330_2021_7882_MOESM1_ESM.docx]

**SUPPLEMENTARY MATERIAL**

**Table S1. Patient-based analysis of RCA-based PCAT_MA_ with CAC score and degree of stenosis categories**

| **Categories** | **RCA PCAT_MA_** | **p-value** |
| --- | --- | --- |
| **Patient status** |  | 0.416 |
| **Patients without plaque** | -95.7±9.6HU (n=72) |  |
| **Patients with plaque** | -94.5±9.5HU (n=93) |  |
| **Category of CAC score** |  | 0.293 |
| **0** | -95.5±9.6HU (n=78) |  |
| **1-99** | -95.1±9.6HU (n=35) |  |
| **100-399** | -96.0±8.9HU (n=34) |  |
| **>400** | -91.0±9.8HU (n=18) |  |
| **Degree of stenosis** |  | 0.379 |
| **No plaque** | -95.7±9.6HU (n=72) |  |
| **DS 1-24%** | -97.0±9.5HU (n=16) |  |
| **DS 25-49%** | -94.0±9.0HU (n=33) |  |
| **DS 50-69%** | -91.1±8.4HU (n=16) |  |
| **DS 70-100%** | -95.6±10.5HU (n=28) |  |

DS is diameter stenosis; CAC is coronary artery calcium; PCAT_MA_ pericoronary adipose tissues mean attenuation.

**Table S2. Correlation between total plaque burden, LRNC burden and patient-based PCAT_MA_**

| **Patient-based PCAT_MA_** | **Plaque burden** | **LRNC burden** |
| --- | --- | --- |
| **Measured at proximal RCA** | -0.170 (p=0.102) | -0.300 (p=0.003) |
| **Mean of proximal PCAT_MA_ of three main vessels** | -0.092 (p=0.383) | -0.257 (p=0.013) |

PCAT_MA_ pericoronary adipose tissues mean attenuation; LRNC lipid-rich necrosis core; RCA right coronary artery.

**Table S3. Proximal PCAT_MA_ models including all vessels with and without plaque**

| **Categories** | **Basic models** | | **Advanced models** | | | |
| --- | --- | --- | --- | --- | --- | --- |
|  | Estimate fixed effect (95%CI) | Estimated mean  (95%CI) (HU) | p-value | Estimate  Fixed effect (95%CI) | Estimated mean  (95%CI) (HU) | p-value |
| **No-plaque** | 0(Ref) | -96.0(-97.4; -94.7) | 0.223* | 0(Ref) | -97.9(-100.5; -95.2) | 0.140* |
| **Non-calcified** | 2.5(-1.2;6.2) | -92.7(-95.7; -89.7) | 0.036 | 3.0(-0.7;6.8) | -94.2(-97.8; -90.5) | 0.020 |
| **Mixed** | 4.2(0.1;8.4) | -95.3(-98.0; -92.6) | 0.637 | 4.3(0.2;8.5) | -97.0(-100.3; -93.6) | 0.549 |
| **Calcified** | 3.2 (0.2;6.3) | -95.4(-97.1; -93.6) | 0.524 | 3.5(0.5; 6.6) | -96.9(-99.6; -94.2) | 0.343 |
|  |  |  |  |  |  |  |
| **No-plaque** | 0(Ref) | -96.0(-97.4; -94.7) | 0.611* | 0(Ref) | -97.9(-100.6; -95.2) | 0.429* |
| **1-24%** | 2.8(-0.6;6.1) | -95.1(-97.4; -92.9) | 0.102 | 3.2(-0.1;6.5) | -96.7(-99.8; -93.6) | 0.328 |
| **25-49%** | 3.6(0.2;7.0) | -94.9(-96.9; -92.9) | 0.038 | 3.8(0.5;7.2) | -96.4(-99.3; -93.5) | 0.194 |
| **50-69%** | 4.4(-3.1;11.8) | -95.7(-99.1; -92.2) | 0.251 | 4.1(-3.3;11.5) | -97.3(-101.3; -93.3) | 0.755 |
| **70-100%** | 3.7(-1.3;8.7) | -93.6(-96.5; -90.7) | 0.149 | 4.3(-0.8;9.3) | -95.1(-98.6; -91.5) | 0.074 |
|  |  |  |  |  |  |  |

CI is confidence interval; HU is Hounsfield unit; PCAT_MA_ pericoronary adipose tissues mean attenuation; p-value with * is the fixed effect p value for the factor.
